# Retrospective study of intrapartum fever in term pregnancies and adverse obstetric and neonatal outcomes

**DOI:** 10.7717/peerj.14242

**Published:** 2022-10-27

**Authors:** Hongmin An, Wei Zheng, Qinghua Zhu, Haiyan Wen

**Affiliations:** 1 Obstetrics Department, Hangzhou Women’s Hospital, Hangzhou, China; 2 Department of Gastroenterology, Children’s Hospital, Zhejiang University School of Medicine, National Clinical Research Center for Child Health, National Children’s Regional Medical Center, Hangzhou, China

**Keywords:** Intrapartum fever, Term pregnancy, Adverse neonatal outcomes, Cesarean delivery, Histological chorioamnionitis

## Abstract

**Background:**

Intrapartum fever is a well-known predisposing factor for severe perinatal outcomes. Herein, we explored the intrapartum features, obstetric outcomes, and neonatal outcomes in relation to the extent of intrapartum fever *via* three group analyses.

**Methods:**

A retrospective cohort analysis consisting of 575 term, singleton live births in one medical center from January 1st to December 31st, 2020 was carried out. Parturients who had experienced a maximal intrapartum fever of <38.0 °C were compared with two sub-groups of parturients who had experienced respective maximal fevers of 38.0–38.9 °C and ≥39.0 °C. We computed the adjusted risks for adverse perinatal outcomes *via* multiple logistic regression models to control for confounders.

**Results:**

There were statistically remarkable differences among the three groups in 13 items including body mass index, epidural, and WBC before delivery (*p* < 0.05). In contrast with intrapartum fevers of 37.5–37.9 °C, intrapartum fevers of 38.0–38.9 °C were linked to an elevated risk of neonatal sepsis and neonatal intensive care unit admission with an odds ratio (OR) of 4.28 (95% CI 2.162–8.479) and 1.73 (95% CI 1.125–2.666), nonetheless, the relationship was remarkably higher for intrapartum fever ≥39.0 °C, with an OR of 6.40 (95% CI 2.450–16.725) and 2.23 (95% CI 1.021–4.854). Additionally, intrapartum fevers of 38.0-38.9 °C and ≥39.0 °C were related to remarkably higher risk for operative deliveries (OR 2.24, 95% CI 1.373–3.648; OR 3.59, 95% CI 1.398–9.226; respectively) and histological chorioamnionitis (OR 3.77, 95% CI 2.261-6.271; OR 19.24, 95% CI 7.385–50.111, respectively).

**Conclusions:**

Intrapartum fever is an important indicator of adverse perinatal outcomes. The higher the temperature, the higher risk of histological chorioamnionitis, as well as the risk of neonatal sepsis and neonatal intensive care unit admission.

## Introduction

Intrapartum fever, often defined as fever ≥ 38.0  °C, may be associated with noninfectious or infectious etiology and is a well-known predisposing factor for perinatal adverse outcomes, consisting of neonatal sepsis, asphyxia, encephalopathy, *etc* (Petrova et al., 2001; Lange et al., 2017; Li, Yang & Zhang, 2021). Severe obstetric outcomes consist of Cesarean delivery, post-partum hemorrhage, dystocia, as well as assisted vaginal delivery (Dior et al., 2016). The incidence of intrapartum fever is between 3.3%∼7% for all deliveries (Braun et al., 2016; Burgess et al., 2017).

There is increasing evidence that most intrapartum fevers arise from non-infectious etiologies, notably epidural analgesia (Riley et al., 2011; Hochler et al., 2021). Nonetheless, notwithstanding its cause, intrapartum fever is linked to severe maternal, as well as neonatal outcomes (Hochler et al., 2021). However, in some investigations, there was no remarkable link between intrapartum fever and long-term adverse events in offspring (Towers et al., 2017). Therefore, the relationship between intrapartum fever at term and infection remains controversial (Kim et al., 2021).

Previous studies on intrapartum fever were mostly based on a fever of ≥ 38.0 °C or <38.0 °C, and often, ≥ 37.5 °C or <37.5 °C has also been utilized (Sharpe & Arendt, 2017). Maternal intrapartum fever ≥ 39.0 °C is a rare event that happens in labor and there is little evidence of its impact. With this background, we purposed to elucidate the relationship of intrapartum fever at term with obstetric, as well as neonatal outcomes. Herein, intrapartum fever was stratified into three classes based on two reference values of 38.0 °C and 39.0 °C. Maternal along with intrapartum features, neonatal outcomes, and obstetric outcomes were compared among the three groups.

## Materials and Methods

### Patients and data selection

**Figure 1:**
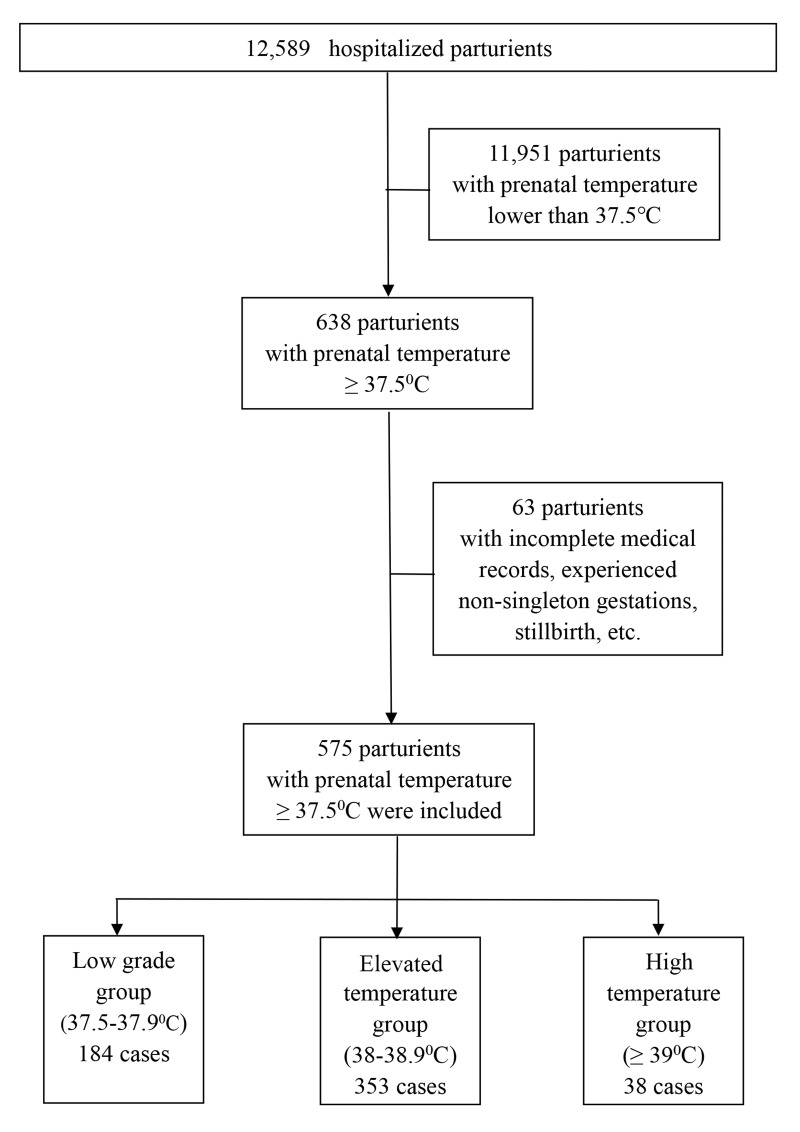
Flow diagram of the parturients that were invited to participate in the study.

Hangzhou Women’s Hospital is a tertiary obstetrics and gynecology specialist hospital directly under the Hangzhou Municipal Health Commission and is an obstetrics and gynecology hospital affiliated with Hangzhou Normal University. It has the important task of guiding the management of maternity and child health care in the whole Hangzhou area, with an annual delivery capacity of approximately 12,000. A retrospective cohort study involving neonates born to mothers suffering from intrapartum fever in Hangzhou Women’s Hospital was performed from January 1st to December 31st, 2020. The medical records of the subjects between 37^0/7^ and 41^0/7^ gestational age who experienced active labor (induced or spontaneous) and experienced a systemic fever of ≥ 37.5 °C along with the records of their respective neonates were reviewed retrospectively. Subjects whose medical records were incomplete, experienced non-singleton gestations, stillbirths, scheduled cesarean section, infants delivered before 37^0/7^ gestational age, or congenital fetal anomalies were not included in this analysis. This study was granted approval by the Medical Ethics Committee of Hangzhou Women’s Hospital (2022K10-05). The oral temperature of the parturients’ is measured routinely before entering the labor room, as well as every eight hours thereafter, when normal. When the oral temperature measured ≥ 37.5 °C, additional temperature measurements followed in the next hour. We stratified the subjects based on maximal oral temperature recorded in labor into subjects harboring maximal temperature ≥ 37.5–37.9 °C (low-grade group; LGG), subjects with a maximal fever of 38-38.9 °C (elevated temperature group; ETG), and those exhibiting maximal fever ≥ 39 °C (high-temperature group; HTG) (Dior et al., 2016; Hochler et al., 2021). The procedures are summarized in the flow diagram (Fig. 1). Data consisting of maternal along with intrapartum manifestations, neonatal outcomes, as well as obstetric outcomes were acquired *via* reviews of the electronic medical records in our institution. Strengthening the Reporting of Observational Studies in Epidemiology (STROBE) guidelines were followed (Cuschieri, 2019).

### Data collection of baseline characteristics

The maternal baseline features and intrapartum manifestations consisted of maternal age, parity, gestational age, gender, body mass index (BMI), education, epidural analgesia, white blood cell (WBC) before delivery, C-reactive protein (CRP) before delivery, prothrombin time (PT) before delivery, activated partial thromboplastin time (APTT) before delivery, peak fever temperature, premature rupture of membranes (PROM) ≥ 18 h, meconium-stained amniotic fluid III (MSAF III), the length of the first stage, second stage and third stage of labor, WBC after delivery, CRP after delivery, gestational hypertension, gestational diabetes mellitus and hypothyroidism, which is linked to intra-uterine temperature in labor (Cheng et al., 2007).

### Diagnosis of neonatal outcomes

The neonatal outcomes evaluated consisted of birth weight, low 1-min Apgar scores (<7), NICU (neonatal intensive care unit) admissions, neonatal asphyxia along with neonatal sepsis. We defined cases of neonatal sepsis as those diagnosed within one-week post-birth, and pediatricians diagnosed suspected sepsis on the clinical findings, for instance, instability of temperature, hypotension, bradycardia, or tachycardia. Determination of cases of proven sepsis was done after isolation of a pathogen from body fluids, for instance, blood or cerebrospinal fluid (Kim et al., 2021).

### Diagnosis of obstetric outcomes

Our obstetric outcomes were assisted vaginal delivery, histological chorioamnionitis, and primary cesarean section. Reviewing of the results of histological chorioamnionitis (HCA) was done by a single pathologist specialist in placental pathology during the period of the study based on the Redline criteria, which had was previously defined, as well as adopted by the Society for Pediatric Pathology (Redline, 2012; Lee et al., 2015).

### Statistical analysis

According to the formula (sample size *n* = C^2^ *σ*^2^/p^2^), the sample size was calculated with reference to the incidence of fever. Previous studies suggest that the rate of intrapartum fever is 3.3%∼7% (Braun et al., 2016; Burgess et al., 2017). As a tertiary obstetrics and gynaecology hospital, our hospital has an annual delivery capacity of approximately 12,000. Inclusion of one year’s worth of patients with antenatal fever is sufficient to study the required sample size. Statistical analyses were implemented in SPSS v20.0 (IBM, Hangzhou, China). Demographics along with clinical features of the study subjects were assessed *via* univariate analysis. *T*-tests and the Mann–Whitney *U*-test coupled with Fisher’s exact test were employed to establish significance, where applicable. The selection of variables was based on previous reports in the literature and also took into account clinical plausibility. Based on the findings from univariate analyses, *p* < 0.05 was set as the threshold for inclusion in Logistic regression analyses. We entered the associated predictors into a binary logistic regression model for identification of the independent relationships and to compute the odds ratios (OR), which were reported with 95% confidence intervals (CI). Logistic regression modeling yielded adjusted odds ratios of the variables possibly linked to operative delivery or severe neonatal outcome while controlling for prospective confounders. All *P*-values were two-tailed, with *p* < 0.05 signifying statistical significance.

## Results

### Patient demographics and clinical data

Among 575 births included in this research premise during the one year of research, 184 parturients (32.0%) harbored maximal temperature in the labor period <38.0 °C (LGG), 353 parturients (61.4%) harbored fever (i.e.a maximal fever of 38.0∼38.9 °C in the labor period) (ETG), and 38 parturients (6.6%) exhibited maximal fever of ≥ 39.0  °C (HTG) in the labor period. The clinical features of the subjects are given in Table 1 showing maternal age, parity, gestational age, *etc*. Parturients with HTG were more likely than their counterparts in the other groups have higher rates of epidural analgesia and lower BMI (84.2 *vs* 64.7 *vs* 78.2% and 23.38 ± 4.01 *vs* 24.94 ± 3.72 *vs* 23.59 ± 4.25, respectively, *p* < 0.05). Clinical outcomes and lab results of the study population are presented in Table 2. There were statistically remarkable differences among the three groups in WBC before delivery, CRP before delivery, peak fever temperature, the second stage of labor, WBC after delivery, CRP after delivery, antipyretic treatment, and antibiotics treatment (*p* < 0.05). Further statistical analysis of the prenatal and postnatal indicators in each group, the results indicated that there was no significant difference between the prenatal WBC and corresponding postnatal WBC in the three groups. The postnatal CRP values were significantly higher than the corresponding prenatal CRP in the three groups.

**Table 1:** Characteristics of parturients by intrapartum temperature.

Characteristic	LGG (*n* = 184)(%)	ETG (*n* = 353)(%)	HTG (*n* = 38)(%)	*p*-value
Maternal age				0.31
20–34 years	176 (96.2%)	345 (97.7%)	36 (94.7%)	
35–45 years	8 (3.8%)	8 (2.3%)	2 (5.3%)	
Parity				0.62
1	176 (96.2%)	346 (98.0%)	37 (97.4%)	
≥ 2	8 (3.8%)	7 (2.0%)	1 (2.6%)	
Gestational age				0.29
37–39	105 (57.1%)	183 (51.8%)	17 (44.7%)	
≥ 40	79 (42.9%)	170 (48.2%)	21 (55.3%)	
Gender (Male)	88 (47.8%)	204 (57.8%)	24 (63.2%)	0.05
BMI: (mean ±SD)	24.94 ± 3.72	23.59 ± 4.25	23.38 ± 4.01	0.001^**^
Education				0.982
Master and Doctor	13 (7.1%)	22 (6.2%)	3 (7.9%)	
University graduate	159 (86.4%)	305 (86.4%)	32 (84.2%)	
Trade school	12 (6.5%)	26 (7.4%)	3 (7.9%)	
Birth weight				0.28
<2,500 g	1 (0.5%)	3 (0.8%)	0 (0.0%)	
2,500–3,499 g	119 (64.7%)	189 (53.5%)	17 (44.7%)	
3,500–3,999 g	54 (29.3%)	126 (35.7%)	18 (47.4%)	
≥ 4,000 g	10 (5.4%)	35 (10.0%)	3 (7.9%)	
Epidural analgesia	119 (64.7%)	276 (78.2%)	32 (84.2%)	0.001^**^

**Notes.**

LGG low grade group ETG eleviated temperature group HTG high temperature group BMI body mass index

* *p* < 0.05; ** *p* < 0.01

**Table 2:** Characteristics before and after delivery by intrapartum temperature.

Characteristic	LGG(*n* = 184)(%)	ETG(*n* = 353)(%)	HTG(*n* = 38)(%)	*p*-value
WBC before delivery: (mean ±SD)				
(×10*^9^/L)	11.89 ± 5.65	14.63 ± 3.53	14.80 ± 4.26	<0.0001^**^
WBC after delivery: (mean ±SD)				
(×10*^9^/L)	12.56 ± 2.98	13.70 ± 3.68	16.05 ± 5.20	<0.0001^**^
CRP before delivery: median (IQR), mg/L				
	5.47 (2.64, 12.98)	13.01 (6.34, 27.34)	15.45 (7.27, 30.68)	<0.0001^**^
CRP after delivery: median (IQR), mg/L				
	62.78 (39.99, 91.89)	91.98 (61.04, 124.86)	123.19 (79.84, 160.00) <	0.0001^**^
PT before delivery: median (IQR), s				
	10.70 (10.20, 11.10)	10.80 (10.30, 11.30)	10.60 (10.18, 11.23)	0.49
APTT before delivery: median (IQR), s				
	25.65 (24.23, 27.00)	25.80 (24.40, 27.10)	26.00 (24.45, 27.90)	0.28
PROM ≥18 h	39 (21.2%)	87 (24.6%)	4 (10.5%)	0.12
MSAF III	33 (17.9%)	65 (18.4%)	6 (15.8%)	0.92
First stage of labor, min				
(mean ±SD)	628.35 ± 246.42	647.82 ± 262.21	743.73 ± 393.63	0.12
Second stage of labor, min				
(mean ±SD)	83.37 ± 54.37	97.69 ± 56.74	99.86 ± 47.32	0.03^*^
Third stage of labor, min				
(mean ±SD)	9.26 ± 5.56	8.62 ± 4.89	9.27 ± 4.10	0.43
Gestational hypertension	11 (6.0%)	18 (5.1%)	0 (0.0%)	0.31
Gestational diabetes mellitus	23 (12.5%)	41 (11.6%)	5 (13.2%)	0.93
Thyroid disease during pregnancy				
Hypothyroidism	28 (15.2%)	46 (13.0%)	6 (15.8%)	0.74
Anti-pyretic treatment	107 (58.2%)	353 (100%)	8 (100%)	0.0001^**^
Antibiotics treatment	84 (45.7%)	262 (74.2%)	32 (84.2%)	<0.0001^**^

**Notes.**

LGG low grade group ETG eleviated temperature group HTG high temperature group WBC white blood cell CRP C-reactive protein PT Prothrombin time APTT activated partial thromboplastin time PROM premature rupture of membranes HCA histological chorioamnionitis NICU neonatal intensive care unit MSAF III meconium-stained amniotic fluid III

* *p* < 0.05; ** *p* < 0.01

### Obstetrical and neonatal outcomes across study groups

The relationships of LGG, ETG, and HTG with obstetric, as well as neonatal outcomes are given in Table 3. There were statistically remarkable differences among the three groups in NICU admission, neonatal sepsis, assisted vaginal delivery, HCA, and primary cesarean section (*p* < 0.05). The incidence of HCA was higher in HTG than in the ETG and LGG (73.7% *vs.* 45.6% *vs.* 13.6%, *p* < 0.0001). Similarly, Parturients with HTGs were more likely than their counterparts in the ETG and LGG to have higher rates of NICU admission (44.7% *vs.* 22.4%, *p* = 0.001). The incidence of neonatal sepsis was higher in HTG than in the ETG and LGG (36.8% *vs.* 24.4% *vs.* 6.0%, *p* < 0.0001). When exploring the association of intrapartum febrile temperature with the extents of severe obstetric, as well as neonatal outcomes in the ETG and HTG groups, a temperature-level response impact was evident in primary cesarean deliveries, HCA, NICU admissions, and neonatal sepsis (Table 3).

**Table 3:** Obstetrical and neonatal outcomes at different temperatures.

Outcome	LGG(*n* = 184) (%)	ETG(*n* = 353) (%)	HTG(*n* = 38) (%)	*p*-value
1-min Apgar score <7	3 (1.7%)	11(3.1%)	3 (7.9%)	0.11
NICU admission	43 (23.4%)	134 (38.0%)	17 (44.7%)	0.001^**^
Neonatal asphyxia	3 (1.7%)	11 (3.1%)	3 (7.9%)	0.11
Neonatal sepsis	11 (6.0%)	86 (24.4%)	14 (36.9%)	<0.001^**^
Positive blood culture	1 (0.5%)	13 (3.7%)	4 (10.5%)	<0.001^**^
Assisted vaginal delivery	10 (5.4%)	42 (11.9%)	9 (23.7%)	0.002^**^
HCA	25 (13.6%)	161 (45.6%)	28 (73.7%)	<0.001^**^
Primary cesarean section	43 (23.4%)	155 (43.9%)	20 (52.6%)	<0.001^**^

**Notes.**

LGG low grade group ETG eleviated temperature group HTG high temperature group NICU neonatal intensive care unit HCA Histological chorioamnionitis

* *p* < 0.05; ** *p* < 0.01

### The association of intrapartum fever with adverse obstetric and neonatal outcomes

The relationships of intrapartum temperature groups with severe obstetric, as well as neonatal outcomes were explored *via* multivariable logistic regressions in Table 4. Models studying obstetric outcomes (either assisted vaginal delivery, HCA, or cesarean section) controlled for BMI, epidural analgesia, WBC before delivery, CRP before delivery, and second stage of labor. Models for neonatal outcomes controlled for BMI, Epidural, WBC before delivery, CRP before delivery, and second stage of labor. Women who had either ETG or HTG experienced similarly elevated risks for either assisted vaginal deliveries or primary cesarean deliveries or HCA. Women with ETG had an OR of 3.77 (95% CI: 2.2613, 6.271) for HCA, and women with HTG had an OR of 19.24 (95% CI: 7.385, 50.111) (*p* = 0.0001 and *p* = 0.0001, respectively). Logistic analysis showed that ETG was significantly linked to neonatal sepsis with an OR of 4.28 (95% CI: 2.162, 8.479); nonetheless, the relationship was significantly higher for HTG, with an OR of 6.40 (95% CI: 2.450, 16.725) (*p* = 0.0001 and *p* = 0.0001, respectively). ETG was significantly associated with NICU admissions with an OR of 1.73 (95% CI: 1.125, 2.666); nevertheless, the relationship was significantly higher for HTG, with an OR of 2.23 (95% CI: 1.021, 4.854) (*p* = 0.013 and *p* = 0.044, respectively).

**Table 4:** The associations of ETG and HTG with obstetric and neonatal outcomes by multiple regression analysis.

Outcome	ETG (*n* = 353)			HTG (*n* = 38)		
	OR	95%CI	*p*-value	OR	95%CI	*p*-value
NICU admission	1.73	1.125, 2.666	0.013^*^	2.23	1.021, 4.854	0.044^*^
Neonatal sepsis	4.28	2.162, 8.479	0.0001^**^	6.40	2.450, 16.725	0.0001^**^
Assisted vaginal						
Delivery	2.03	1.253, 4.341	0.036^*^	9.43	2.821, 31.506	0.0001^**^
HCA	3.77	2.261, 6.271	0.0001^**^	19.24	7.385, 50.111	0.0001^**^
Primary Cesarean						
Section	2.24	1.373, 3.648	0.001^**^	3.59	1.398, 9.226	0.008^**^

**Notes.**

ETG eleviated temperature group HTG high temperature group NICU neonatal intensive care unit HCA Histological chorioamnionitis

* *p* < 0.05; ** *p* < 0.01

## Discussion

Maternal temperature increases as a result of inflammation and other than bacterial infections, it may be influenced by various factors for instance the activity along with the intensity of uterine contractions, as well as physiological changes, consisting of the increase in basal metabolic rates (Anim-Somuah et al., 2018; Jiang et al., 2021). Other etiologies of intrapartum fever consist of antepartum infections, for instance, urinary/respiratory tract infections, and drugs among others (Gupta et al., 2021; Ren et al., 2021). Antepartum and intrapartum manifestations offer remarkable information concerning exposure to infectious diseases, which informs neonatologists of obstetric risk factors for neonatal infections (Report of the American College of Obstetricians and Gynecologists task force on neonatal encephalopathy, 2014; Fan et al., 2021). Intrapartum fever is monitored to prevent the transfer of infections to newborns with immature immune systems.

In this retrospective cohort research investigation, the results indicated that BMI values of pregnant women in the LGG group were significantly higher than those in the ETG and HTG groups. The relationship between BMI and intrapartum fever is less well studied. Using multivariate logistic regression analysis, Harrison, Egede & Palatnik (2021) found no association between BMI, maternal age, number of births and gestational age at delivery and perinatal infection. Deshmukh, Jadhav & Yelikar (2016) reported that postpartum fever and prolonged hospital stay were strongly associated with raised BMI, whereas intrapartum fever was not associated with BMI. Combined with previous studies we concluded that there was no evidence of correlation between BMI and intrapartum fever, and our results only indicate a statistical difference in BMI between the three groups, with no corresponding clinical significance.

Our results showed that pregnant women receiving epidural analgesia in the HTG group were significantly higher than those in the LGG and ETG groups, indicating that the use of epidural analgesia during labor was associated with intrapartum maternal fever. This was consistent with previous reports (Yin & Hu, 2019; Zhao et al., 2021). Zhao et al. reported that fever after epidural analgesia was not associated with neonatal complications (Zhao et al., 2021). A further study by Yin et al. found that epidural anesthesia did not influence on the mode of delivery, the amount of post-partum hemorrhage or hospital stay after delivery, and all the neonatal outcomes (Yin & Hu, 2019). Our results showed that the incidence of HCA was higher in HTG than in the ETG and LGG (73.7% *vs.* 45.6% *vs.* 13.6%). HCA is also known as subclinical chorioamnionitis, frequently exhibits no symptoms, with raised body temperature being the sole clinical indication in the majority of the symptomatic cases and can diagnosed only by pathological investigation of the placenta (Tita & Andrews, 2010; Ganer Herman et al., 2019). Although the clinically diagnosed chorioamnionitis had typical symptoms and signs, only half of them were confirmed with histologic evidence. Our findings confirm the correlation between HCA and intrapartum fever, and the higher the body temperature, the higher the incidence of HCA.Previous investigations have shown that women experiencing fever are at elevated risk of operative delivery (Lieberman et al., 1997; Petrova et al., 2001). Hochler et al. (2021) found that protracted labor is linked to intrapartum fever. Numerous vaginal assessments were repeatedly documented to escalate the risk of intrapartum maternal fever (Petrova et al., 2001), illustrating that ascending infection from the vagina is among the contributing factors. Lieberman et al. (1997), in an investigation of 1,233 multiparous women harboring singleton term pregnancies, documented 3-fold elevated risks for either CS or aided vaginal deliveries, if the maternal intrapartum fever was ≥ 38 °C. Similar to their findings, we found that the length of phase 2 of labor in HTG was remarkably longer than that in ETG and LGG, and remarkable increased intrapartum fever was also remarkably linked to elevated rates of emergency CSs, operative vaginal deliveries, and HCA with ORs of 3.59, 9.43 and 19.24, respectively. Based on our findings, we propose that in individuals harboring elevated peak temperature or moderate fever for a prolonged duration, the medical team should avoid prolonged labor to decrease the exposure of the fetus to harm. We established that intrapartum fever was linked to severe neonatal outcomes, such that extremely elevated fevers were remarkably linked to 2.23- and 6.40-fold elevated risks of NICU admissions and neonatal sepsis, respectively. Dior et al. explored the relationship of very high intrapartum maternal fever (>39.0  °C) with perinatal outcomes in term pregnancies (Dior et al., 2016). Congruent with our results, remarkably elevated intrapartum fever emerged as a remarkable risk factor for severe neonatal morbidity along with operative delivery.

Previous investigations have documented the significance of maternal inflammatory settings as a primary contributor to intrapartum fever. Smulian et al. (2003) established that inflammation is an indispensable etiologic factor of intrapartum fever *via* exploring high maternal along with umbilical vein serum contents of interleukin 6 in parturients experiencing fever. Given that intrapartum fever is linked to elevated serum contents of inflammatory cytokines in the fetus, as well as the mother, higher or prolonged febrile periods may intensify the inflammatory reaction and be linked to a more severe outcome. Our results exhibited that WBC before delivery and CRP before delivery in HTG were remarkably higher than those in ETG and LGG, the difference was statistically remarkable. This illustrates that elevated maternal fever calls for attention regarding infectious factors. The results have illustrated that the neonatal complications’ odds worsen with increasing intrapartum maternal fever. The association of “temperature-level response” of maximal maternal fever along with the duration with the composite fetal severe outcomes illustrates that elevated maternal fever requires a more active management strategy. When exploring the association of intrapartum febrile temperature with the extents of severe obstetric, as well as neonatal outcomes, a temperature-level response impact was evident, but there is little research on the associated pathogenesis. Intrapartum fever may lead to elevated levels of pyrogens such as PGE2 or PGF2A and inflammatory cytokines such as IL-1 *β*, IL-6 and IL-8 (Abramov et al., 2004). These cytokines were associated with dose–response effects, where higher cytokine levels were associated with worse outcomes (Chiesa et al., 2003), a field that has been substantially studied, especially in the preterm neonatal population (Carlo et al., 2011). Since extremely high fever before labor may be caused in part by extremely high cytokine levels, it may be a useful clinical signal indicating vulnerability to adverse outcomes in this condition.

This research has numerous strengths, as well as drawbacks. A large-sized cohort was collected and analyzed. The correlation between HCA and intrapartum fever was confirmed, while the higher the body temperature, the higher the incidence of HCA. This research premise addressed intrapartum fever temperatures along with perinatal outcomes as the primary outcomes. The primary drawback of our research lies in its retrospective aspect with its setbacks, for instance, potential data bias. To reduce this bias, cases were screened, as well as reviewed meticulously. Due to the very small number of outcome variables in some groups, the small number of cases may interfere with the results of the statistical analysis. Since all cases were selected and were included from Hangzhou Women’s Hospital, there is a likelihood of results harboring confounding variables, which are unavoidable in all single-centered investigations. In view of that, result from large-scale multicenter prospective clinical studies is recommended.

Notwithstanding its etiology, maternal intrapartum fever harbors risk to the mother, as well as her fetus. The maternal fever magnitude, solely, as well as when integrated with duration, is linked to maternal along with neonatal peripartum severe events.

## Conclusions

Intrapartum fever is an important indicator of adverse perinatal outcomes. The higher the temperature, the higher risk of histological chorioamnionitis, as well as the risk of neonatal sepsis and neonatal intensive care unit admission.

##  Supplemental Information

10.7717/peerj.14242/supp-1 Table S1 WBC before and after delivery by intrapartum temperature

10.7717/peerj.14242/supp-2 Table S2 CRP before and after delivery by intrapartum temperature

10.7717/peerj.14242/supp-3 Data S1 Raw data

## Additional Information and Declarations

### Data Availability

The following information was supplied regarding data availability: The raw data are available in the Supplementary Files.
