# Supplementary material for: Retrospective study of intrapartum fever in term pregnancies and adverse obstetric and neonatal outcomes
Source: PeerJ. 2022 Oct 27;10:e14242. doi: 10.7717/peerj.14242 (PMC9618262; doi:10.7717/peerj.14242)
Supplement: Table S1 [file peerj-10-14242-s001.docx]

**Table S1.** WBC before and after delivery by intrapartum temperature

| Groups WBC before delivery: WBC after delivery : *p*-value  (mean±SD) (×10*^9^/L) (mean±SD) (×10*^9^/L) |
| --- |

LGG (n= 184) 11.89±5.65 12.56±2.98 0.152

ETG (n=353) 14.63±3.53 13.70±3.68 0.126

HTG (n= 38) 14.80±4.26 16.05±5.20 0.169

| LGG: low grade group; ETG: eleviated temperature group; HTG: high temperature group; WBC: white blood cell. |
| --- |
