# Supplementary material for: Retrospective study of intrapartum fever in term pregnancies and adverse obstetric and neonatal outcomes
Source: PeerJ. 2022 Oct 27;10:e14242. doi: 10.7717/peerj.14242 (PMC9618262; doi:10.7717/peerj.14242)
Supplement: Table S2 [file peerj-10-14242-s002.docx]

**Table S2.** CRP before and after delivery by intrapartum temperature

| Groups CRP before delivery: CRP after delivery : *p*-value  median (IQR), mg/L median (IQR), mg/L |
| --- |

LGG (n= 184) 5.47 (2.64, 12.98) 62.78 (39.99, 91.89) 0.0001 **

ETG ( n=353) 13.01 (6.34, 27.34) 91.98 (61.04, 124.86) 0.0001 **

HTG (n= 38) 15.45 (7.27, 30.68) 123.19 (79.84, 160.00) 0.0001 **

| LGG: low grade group; ETG: eleviated temperature group; HTG: high temperature group; CRP: C-reactive protein. |
| --- |

**p* < 0.05; ***p* < 0.01
